# Recessive Loci *Pps-1* and *OM* Differentially Regulate *PISTILLATA-1* and *APETALA3-1* Expression for Sepal and Petal Development in *Papaver somniferum*

**DOI:** 10.1371/journal.pone.0101272

**Published:** 2014-06-30

**Authors:** Sharad K. Singh, Ashutosh K. Shukla, Om P. Dhawan, Ajit K. Shasany

**Affiliations:** 1 Genetics and Plant Breeding Division, CSIR-Central Institute of Medicinal and Aromatic Plants, Lucknow, Uttar Pradesh, India; 2 Biotechnology Division, CSIR-Central Institute of Medicinal and Aromatic Plants, Lucknow, Uttar Pradesh, India; Ohio State University, United States of America

## Abstract

The involvement of PISTILLATA (PI) and APETALA (AP) transcription factors in the development of floral organs has previously been elucidated but little is known about their upstream regulation. In this investigation, two novel mutants generated in *Papaver somniferum* were analyzed - one with partially petaloid sepals and another having sepaloid petals. Progeny from reciprocal crosses of respective mutant parent genotypes showed a good fit to the monogenic Mendelian inheritance model, indicating that the mutant traits are likely controlled by the single, recessive nuclear genes named “*Pps-1*” and “*OM*” in the partially petaloid sepal and sepaloid petal phenotypes, respectively. Both paralogs of *PISTILLATA* (*PapsPI-1* and *PapsPI-3*) were obtained from the sepals and petals of *P. somniferum*. Ectopic expression of *PapsPI-1* in tobacco resulted in a partially petaloid sepal phenotype at a low frequency. Upregulation of *PapsPI-1* and *PapsAP3-1* in the petal and the petal part of partially petaloid sepal mutant and down-regulation of the same in sepaloid petal mutant indicates a differential pattern of regulation for flowering-related genes in various whorls. Similarly, it was found that the recessive mutation *OM* in sepaloid petal mutant downregulates *PapsPI-1* and *PapsAP3-1* transcripts. The recessive nature of the mutations was confirmed by the segregation ratios obtained in this analysis.

## Introduction

The MADS-box gene family encodes a series of transcription factors involved in controlling vegetative development in plants, flowering time and the formation of flowers [1], [2], [3]. Floral organ identity genes were first described in the model angiosperms *Antirrhinum majus* and *Arabidopsis thaliana*, leading to the proposal of the ABC model of flower development [4]. Most of the genes corresponding to these functions, with the exception of *APETALA2*, are members of the MADS-box family of transcription factors [5]. *PISTILLATA* (*PI*) and its homologs are classified as B-class genes of the MADS-box family and function together with another B-class gene, *APETALA3* (*AP3*), by forming heterodimers for regulating petal and stamen development in eudicots [6], [7], [8], [9], [10]. The functions of these genes appear to be conserved across the orthologs analyzed among the core eudicots [8], [11], [12] and monocots [13], [14], [15]. A considerable amount of knowledge is available about the molecular mechanisms specifying petal identity in *Arabidopsis* and other core eudicot species however there is little functional evidence regarding homologs with similar roles in petal-identity specification outside of the core eudicots [16] leading to a significant knowledge gap concerning plant organ differentiation, growth and development outside of the most well-studied model systems.

**Figure 1:**
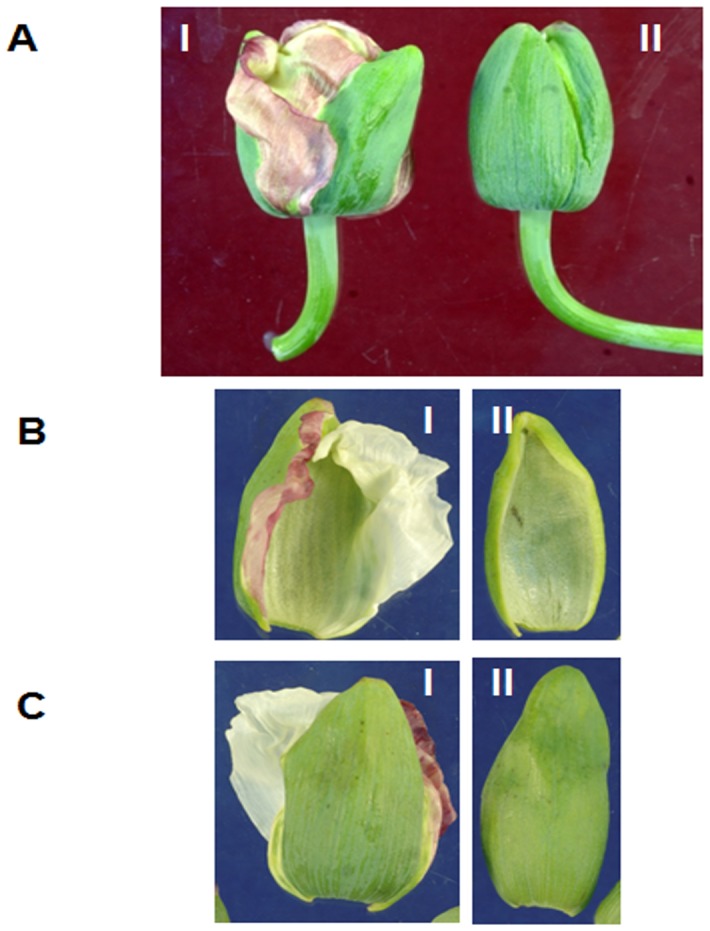
Comparison of floral morphology between the partially petaloid sepal mutant (Pps-1; I) and the parent (I-14; II). A. Flower bud; B. Ventral view of sepal; C. Dorsal view of sepal.

**Figure 2:**
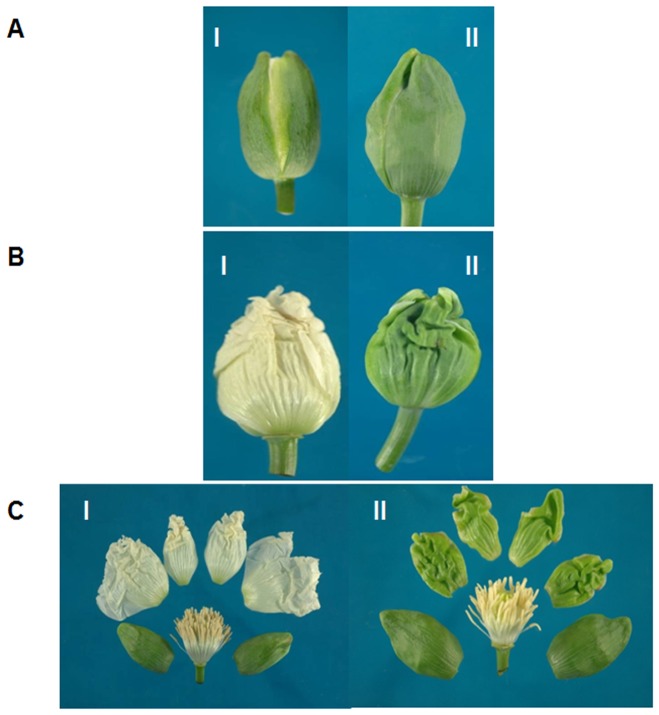
Comparison of floral morphology between the parent I-268 (I) and the sepaloid petal mutant (OM; II). A. Flower bud; B. Flower bud without sepal; C. Dissected sepal and petal.

Opium poppy (*Papaver somniferum*) has a long history of practical, medicinal use spanning thousands of years and it continues to be one of the world's most important medicinal plants due to its unique ability to synthesize the drugs morphine, codeine and thebaine and a variety of other biologically active cyclopentanophenanthrene and benzylisoquinoline alkaloids in its seed pods. Drea et al. [16] described the roles of several MADS-box genes involved in petal specification by demonstrating the duplication and sub-functionalization of *AP3* lineage in *P. somniferum* In poppy, one gene copy influences petal development while the other is responsible for stamen development, contrasting the described role of *AP3* in *Arabidopsis* where *AP3* influences both petal and stamen development. Drea et al. [16] also investigated two paralogs of *PISTILLATA* (*PapsPI-1 and PapsPI-2*) and showed that the *PapsPI-1* gene encodes a product containing the PI-motif as well as a sequence extension at the C-terminus whereas the predicted product of *PapsPI-2* lacks the consensus PI-motif [17] at the C-terminus. This truncation is due to a single nucleotide insertion in the 3' coding region followed by a 2-nucleotide deletion 22 bp downstream that generates a stop codon. Although this domain has been shown to be essential for protein function in *Arabidopsis* PI [18], the *Pisum sativum PI* gene also lacks this conserved domain but has been shown to be capable of rescuing the *Arabidopsis pi*-mutant phenotype [19]. In the present investigation we analyzed different genes involved in flower development by utilizing partially petaloid sepal (Pps-1) and sepaloid petal (OM) mutants that were obtained from the normal sepal and petal phenotypes of I-14 and I-268, respectively. The development of Pps-1 has been described earlier [20], [21]. In the Pps-1 mutant, a part of the sepal is converted into petal rather than forming a complete sepal (**Figure 1**) whereas in OM the whole petal is converted into a sepal (**Figure 2**). These analyses indicate the involvement of different recessive mutations for erroneous interconversion of sepals and petals.

## Materials and Methods

### Plant Material

Plant material consisted of the Pps-1 genotype of *P. somniferum* with partially petaloid sepals, which spontaneously originated from the downy mildew (DM)-resistant genotype I-14. The parent genotype I-14 is characterized by narrow leaves with very deep leaf incisions and white flower petals [20]. In Pps-1, the margins of the sepals are modified into petal-like characters (**Figure 1**). Apart from this, true breeding genotypes I-268 and OM (having mutation ‘*OM*’) were selected to test the hypothesis that specific genes are involved in organ conversion. OM was detected in the open pollinated population of the genotype I-268 of opium poppy in which the petals are converted into sepal-like organs (**Figure 2**). All inbred lines (at least 6 selfing cycles) of mutants and their parents were grown and maintained in the research farm of CSIR-Central Institute of Medicinal and Aromatic Plants, Lucknow, India since 2007 and the true-breeding characters were maintained.

### Breeding

Previously, the segregation of *Pps-1* mutation at a ratio of 3∶1 has been shown [20], confirming the involvement of single recessive gene in regulating the partially petaloid character. For segregation analysis of the second mutant, the parent genotype I-268 having normal white petals was crossed with OM mutant having sepaloid petals. The crossing was carried out normally and reciprocally taking both as male or female parents. The F_1_ and F_2_ generation plants were scored for sepaloid petal character. The collected seeds were sown in the field in randomized block design with 3 replications and observations were collected on a single plant basis. Chi-square analysis was applied to test the goodness-of-fit for frequency distributions in the F_2_ generations (**Table 1**). A 3∶1 segregation of the OM character indicated the involvement of nuclear recessive mutation.

**Table 1:** Segregation pattern of the sepaloid petal (OM) mutant in different generations of the reciprocal crosses involving parent genotype I-268 having wild type phenotype.

Genotype	Characteristics of the genotype	Generation	Number of observed plants		Segregation ratio	Chi-Square	P
			Wild type	Mutant type			
OM	*OM*, homeotic mutant (with green sepaloid petals)	P_1_	0	133			
I-268	Parent genotype from which *OM* mutant evolved (with white petals)	P_2_	106	0	-	-	-
OM×I-268	All plants exhibit white petals with light pink margin	F_1_ (P_1_×P_2_)	110	0	-		-
I-268×OM	All plants exhibit white petals with light pink margin	r F_1_ (P_2_×P_1_)	123	0	-		-
OM×I-268	-	F_2_ (P_1_×P_2_)	82	23	3∶1	0.53	0.50–0.30
I-268×OM	-	r F_2_ (P_2_×P_1_)	56	14	3∶1	1.18	0.30–0.20

### RNA isolation for cDNA preparation and cloning of *PapsPI* gene

Total RNA was isolated from 100 mg of ground tissue samples (from fully developed buds) before anthesis using Trizol reagent (Invitrogen, Cleaveland, OH, USA). RNA was converted into cDNA using the ThermoScript RT-PCR System (Invitrogen, USA) and gene-specific primers [16] were used to amplify the *PapsPI* gene. Amplicons were cloned in pGEM-T easy vector system (Promega) and a total of 20 clones were sequenced for each type of tissue sample (normal sepals of I-14, partially petaloid sepals of Pps-1 and normal petals of both genotypes).

### Phylogenetic analysis

Amino acid sequences were aligned (and phylogeny was reconstructed using Bootstrap maximum likelihood method MEGA5 [22]) with MUSCLE multiple sequence alignment [23], [24].

### Expression analysis using quantitative and semi-quantitative RT-PCR

Quantitative RT-PCR was carried out using SYBR Green chemistry (Applied Biosystems, USA) as described earlier [25]. Gene-specific primers were designed with Primer Express software (*v*2.0; Applied Biosystems, USA) and custom-synthesized from Sigma Aldrich, India. The reactions were carried out in 5 biological replicates on the 7900HT Fast Real Time PCR System (Applied Biosystems, USA) and the specificity of the reactions was verified by melting curve analysis with the thermal cycling parameters: initial hold (50°C for 2 min); initial denaturation (95°C for 10 min); and 40 amplification cycles (95°C for 15 s; and 60°C for 1 min) followed by additional steps (60°C for 15 s, 95°C for 15 s and 37°C for 2 min). Relative mRNA levels were quantified with respect to endogeneous control genes (*actin* [EB740770] in case of *P. somniferum* or ubiquitin [U66264.1] in case of *Nicotiana tabacum*) [26], [27]. Sequence Detection System (S.D.S.) software version 2.2.1 was used for relative quantification of gene transcript using the ΔΔ C_T_ method. Threshold cycle (Ct) values obtained after real time PCR were used for calculation of ΔCt value (target-endogenous control). The quantification was carried out by calculating ΔΔCt to determine the fold difference in gene expression [Δ Ct target - Δ Ct calibrator]. Relative quantity (RQ) was determined by 2^−ΔΔCT^. Semi-quantitative RT-PCR was performed by following the protocol of Misra et al [26]. Primers were designed on the basis of *P. somniferum* (for *PapsPI-1*, *PapsPI-2*, *PapsAP3-1* and *PapsAP3-2*) gene sequences. Details of the primers used in the semi-quantitative RT- PCR have been provided in **Table S1**.

### Tobacco transformation

Specific primers were designed to prepare the overexpression construct for the *PapsPI-1* gene. *Xba*I (forward primer) and *Bam*HI (reverse primer) restriction sites were introduced at either sides of the coding sequence. The amplified PCR-product was cloned in pGEM-T Easy vector and the sequence was confirmed. The plasmid containing the coding region was digested with *Xba*I and *Bam*HI and cloned into pBI121 under the control of the CaMV 35S promoter to yield the final construct *35S*::*PapsPI-1*. Binary vectors with and without the transgene were separately transformed into GV3101 strain of *Agrobacterium* and used to generate transgenic tobacco plants as described [28], [29]. Transformants were observed after 3-4 weeks of selection on kanamycin (200 µg ml^-1^). Regenerated shoots were excised and rooted. Plantlets with well established root system were hardened for 2 weeks, subsequently transferred to soilrite mix (Keltech Energies Limited, India) and irrigated with diluted MS media. Fully acclimatised plantlets were grown in the greenhouse and genomic DNA samples of transgenic tobacco lines were screened by PCR using *NPTII* and *PapsPI-1* specific primers to verify the transfer of transgene cassettes into the transgenic lines. The non-transformed plants and empty vector-transformed plants did not show any amplification (**Figure S1**).

## Results

### Expression patterns for genes involved in flowering

**Figure 3:**
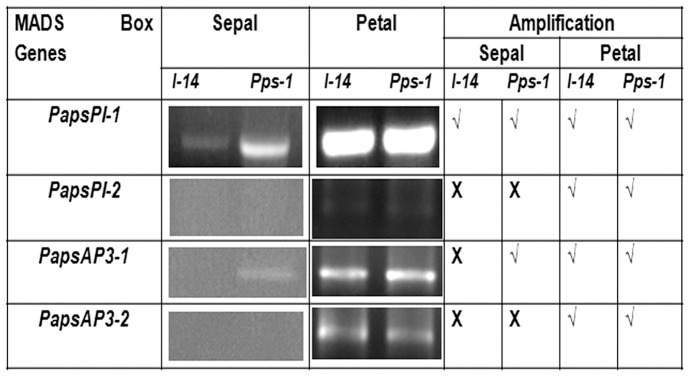
Expression of flowering-related genes in the partially petaloid sepal of Pps-1 and the normal sepal of I-14. *PapsPI-1*: *Papaver somniferum PISTILLATA* 1; *PapsPI-2*: *P. somniferum PISTILLATA* 2; *PapsAP3-1*: *P. somniferum APETALA*3-1; *PapsAP3-2*: *P. somniferum APETALA*3*-*2. Boxes on the right side of the gels indicate amplification (**√**) or no amplification (**X**), denoting the detection/non detection of homologous genes in *P. somniferum*.

Expression level was determined for the four genes of the ABC model in sepals and petals of both the Pps-1 mutant and the wild-type (I-14) through semi-quantitative RT-PCR. Among the genes analyzed, the most significant difference was observed for *PapsPI-1* whose expression was significantly higher in the partially petaloid sepal relative to normal sepal of I-14 (**Figure 3**). Differential expression was not detected in the petals of flowers produced by Pps-1 and I-14. A similar expression pattern was also detected for *PapsAP3-1* (**Figure 3**).

### Cloning of *PapsPI* gene copies/paralogs and phylogenetic analysis

**Figure 4:**
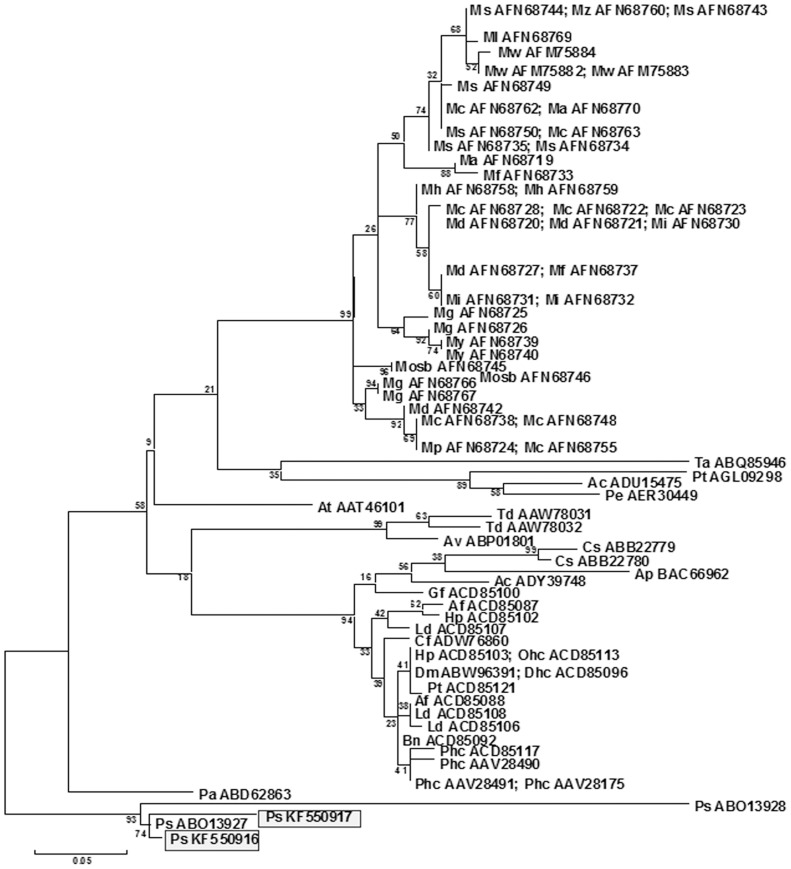
Unrooted maximum likelihood tree comparing the amino acid sequences of *P. somniferum* PI-1(KF550916), with PI-1 (PISTILLATA) reported from other species. The symbols for the plants are provided along with the GenBank accession numbers in brackets. *Agapanthus praecox* Ap (BAC66962); *A. praecox*, Ap (ADU15475); *Akebia trifoliate* At (AAT46101); *Ananas comosus* Ac (ADY39748); *Anoectochilus formosanus* Af (ACD85087); *A. formosanus* Af (ACD85088); *Aquilegia vulgaris* Av (ABP01801); *Brassavola nodosa* Bn (ACD85092); *Crocus sativus* Cs (ABB22779); *C. sativus* Cs (ABB22780); *Cymbidium faberi* Cf (ADW76860); *Dendrobium hybrid cultivar Dhc* (ACD85096); *Dendrobium moniliforme* Dm (ABW96391); *Galeola falconeri* Gf (ACD85100); *Habenaria petelotii* Hp (ACD85102); *H. petelotii* Hp (ACD85103); *Liparis distans* Ld (ACD85106); *Ludisia discolor* Ld (ACD85107); *L. discolor* Ld (ACD85108), *Michelia alba* Ma (AFN68719); *Magnolia amoena* Ma (AFN68770); *M. championii* Mc (AFN68738); *M. championii* Mc (AFN68748); *M. championii* Mc (FN68738); *M. coco* Mc (AFN68755); *M. conifera var. chingii* Mc (AFN68728); *M. crassipes* Mc (AFN68722); *M. crassipes* Mc (AFN68723); *M. cylindrica* Mc (AFN68762); *M. cylindrica* Mc (AFN68763); *M. dandyi* Md (AFN68727); *M. delavayi* Md (AFN68745); *M. duclouxii* Md (AFN68720); *M. duclouxii* Md (AFN68721); *M figo* Mf (AFN68733); *M. fordiana* Mf (AFN68737); *M. grandiflora Mg* (*AFN68766*); *M. grandiflora* Mg (AFN68767); *M. grandis* Mg (AFN68725); *M. hookeri* Mh (AFN68758); *M. hookeri* Mh (AFN68759); *M. insignis* Mi (AFN68730); *M. insignis* Mi (AFN68731); *M. insignis* Mi (AFN68732); *M. liliiflora* Ml (AFN68769); *M*. *officinalis subsp. biloba* Msob (AFN68745); *M. officinalis subsp. biloba* Mosb (AFN68746); *M. paenetalauma* Mp (AFN68724); *M. paenetalaum* Mp (AFN68726); *M. salicifolia* Ms (AFN68734); *M. salicifolia* Ms (AFN68735); *M. sprengeri* Ms (AFN68743); *M. sprengeri* Ms (AFN68744); *M. stellata* Ms (AFN68749); *M. stellata* Ms (AFN68750); *M. wufengenesis* Mw (AFM75882); *M. wufengenesis* Mw (AFM75883); *M. wufengenesis* Mw (AFM75884); *M. yunnanensis* My (AFN68739); *M yunnanensis* My (AFN68740); *M. zenii* Mz (AFN68760); *Oncidium hybrid cultivar* Ohc (ACD85113); *Papaver somniferum* Ps (KF550916); *P. somniferum* Ps (KF550917); *P. somniferum* Ps (ABO13927); *P. somniferum* Ps (ABO13928); *Paphiopedilum* hybrid cultivar Phc (ACD85117); *Passiflora edulis* Pe (AER30449); *Persea americana* Pa (ABD62863); *Phaius tancarvilleae Pt (ACD85121); Phalaenopsis* hybrid cultivar Phc (AAV28175); *P.* hybrid cultivar Phc (AAV28490); *P.* hybrid cultivar Phc (AAV28491); *Populus tomentosa* Pt (AGL09298); *Thalictrum dioicum* Td (AAW78031); *T. dioicum* Td (AAW78032); *Trochodendron aralioids* Ta (ABQ85946).

*PapsPI-1* was cloned from cDNA transcribed from the RNA of normal sepals of I-14, partially petaloid sepals of Pps-1 and normal petals of both genotypes. PCR was carried out using gene-specific primers derived from GenBank sequence EF071994 (amino acid ABO13927) [16]. All sequenced amplicons (20 clones each from sepals and petals of both genotypes) were identical, and the *PapsPI-1* sequence (KF550916) from this investigation was 99% similar to the earlier reported *PapsPI-1* (EF071994) [16]. Interestingly, the present study also generated another copy of the *PapsPI* gene (*PapsPI-3*, deposited under Accession No. F550917) that had a stop codon introduced at the 151 amino acid position due to a single base deletion (adenine). This copy was obtained from partially petaloid sepals (of Pps-1), normal sepals (of I-14) and petal tissues of both I-14 and Pps-1. Additionally, seventeen point mutations were also detected in *PapsPI-3* as compared to *PapsPI-1*, of which, nine were before the stop codon in *PapsPI-3*. In phylogenetic analysis the *PapsPI-1* sequence of this investigation (KF550916) and the one reported earlier (ABO13927) [16] clustered together but the sequence of *PapsPI-2* reported earlier (nucleotide EF071995, amino acid ABO13928) [16] was different from that of *PapsPI-3* reported in this investigation (KF550917; **Figure 4**).

### *PapsPI-1* and *PapsAP3-1* expression in Pps-1 genotype

**Figure 5:**
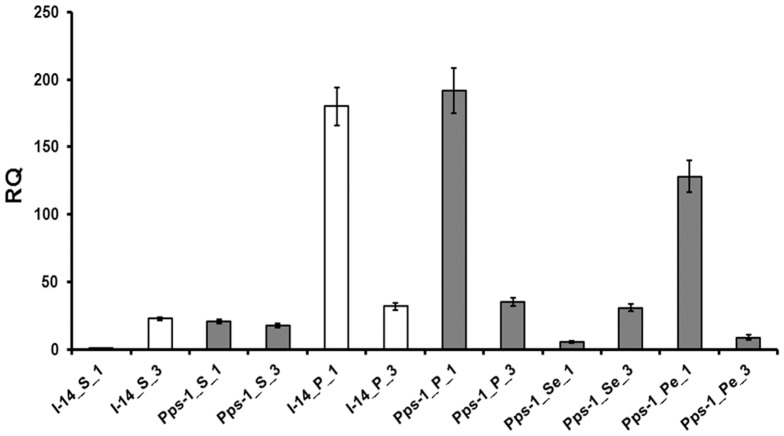
Comparison of quantitative expression levels of *PapsPI-1* and *PapsPI-3* in the petals and sepals of the genotypes Pps-1 and I-14. The Y-axis represents relative quantities equilibrating the expression of *PapsPI-1* in I-14 sepals as 1RQ value. Data represent mean + standard error of 3-5 biological replicates. In X-axis, the names of the genotypes (Pps-1 and I-14) are followed by the organs (S: sepal; P: Petal; Se: Sepaloid part of the sepal and Pe: Petaloid part of the sepal) and *PapsPI* gene expression (1: *PapsPI-1* and 3: *PapsPI-3*). Shaded bar represents Pps-1 genotype.

**Figure 6:**
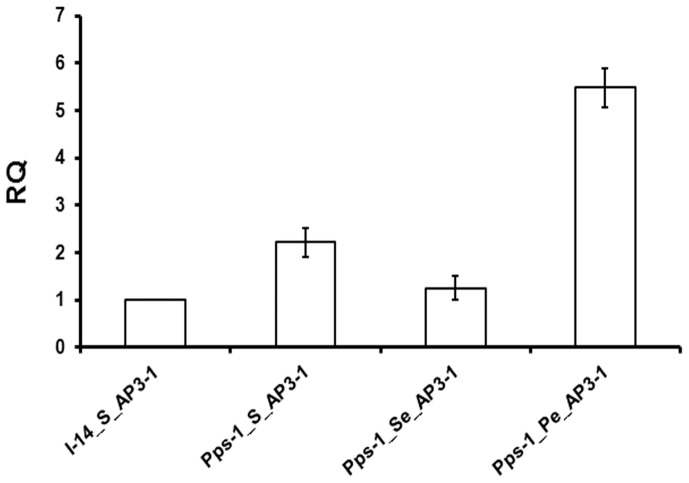
Comparison of quantitative expression levels of *PapsAP3-1* in the sepals of genotypes Pps-1 and I-14. The Y-axis represents relative quantities equilibrating the expression of *PapsAP3-1* in I-14 sepals as 1RQ value. Data represent mean + standard error of 3-5 biological replicates. On X-axis, S: sepal; Se: Petaloid-devoid part of the sepal and Pe: Petaloid part of the sepal.

Higher transcript abundance was observed for *PapsPI-1* in the sepals of Pps-1 as compared to the sepals of I-14. But in the petals of both I-14 and Pps-1, the expression was about 200-fold higher as compared to the sepals of I-14 (**Figure 5**) indicating the causative link between elevated *PapsPI-1* expression and petal tissue specification. When the petaloid part was dissected from the partially petaloid sepal of Pps-1, a 23-fold higher expression for *PapsPI-1* was observed in the petaloid part as compared to the remaining sepal part. Although, a specific trend of expression for *PapsPI-3* was not detected either in the sepal or petal of I-14 and Pps-1, it was interesting to note that higher expression of *PapsPI-3* relative to *PapsPI-1* was observed in the sepal (devoid of petaloid portion) part of Pps-1, as in the case of the true sepal of I-14 (**Figure 5**). Also, in the petaloid part of the Pps-1 sepal and the petals of both I-14 and Pps-1, *PapsPI-3* expression was always found to be lower than that of *PapsPI-1*. *PapsAP3-1* expression was higher in the petaloid part of the partially petaloid sepal of Pps-1 as compared to that in its petaloid-devoid sepal part (**Figure 6**).

### Transformation of *PapsPI-1* in tobacco

**Figure 7:**
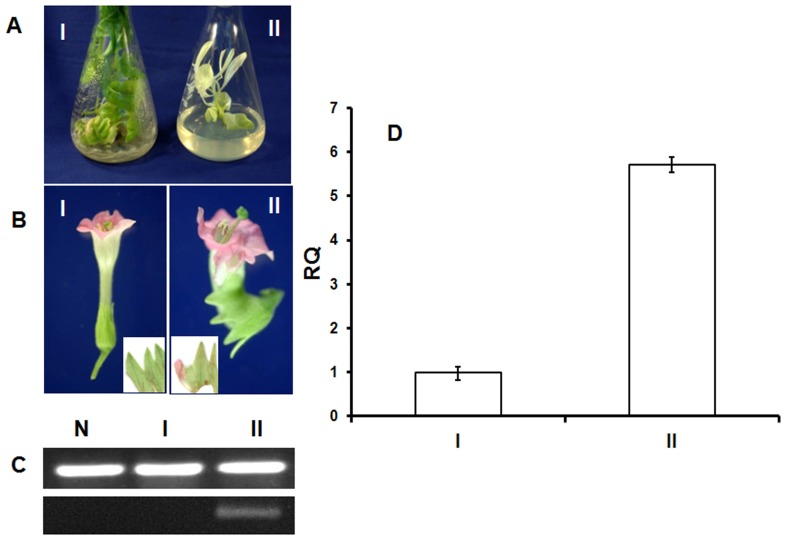
Flower of tobacco plant transformed with *PapsPI-1*. A: Plants in culture (I: Vector transformed; II: Transformed with *PapsPI-1*); B: Flower (I: Vector transformed; II: Transformed with *PapsPI-1*; inset normal sepal and partially petaloid sepal); Semi-quantitative expression of *PapsPI-1* (upper gel shows ubiquitin expression and the lower shows expression of transgene *PapsPI-1*) in: N: Non transformed, I: Vector transformed and plant, II: transformed with *PapsPI-1*); D: Quantitative expression in vector transformed (I) and *PapsPI-1* transformed tobacco sepal (II).

*PapsPI-1* was expressed constitutively in tobacco under the influence of the CaMV 35S promoter. Flowers were obtained in all the twenty transgenic plants screened but only one plant produced a flower having the partially petaloid sepal character (**Figure 7**). Ten plants showed pale green morphology with differential leaf arrangements compared to the control plant. Six-fold increased expression of *PapsPI-1* was observed in the partially petaloid sepal of the transformed flower (**Figure 7D**).

### Segregation of *OM* mutation and *PapsPI-1* expression

**Figure 8:**
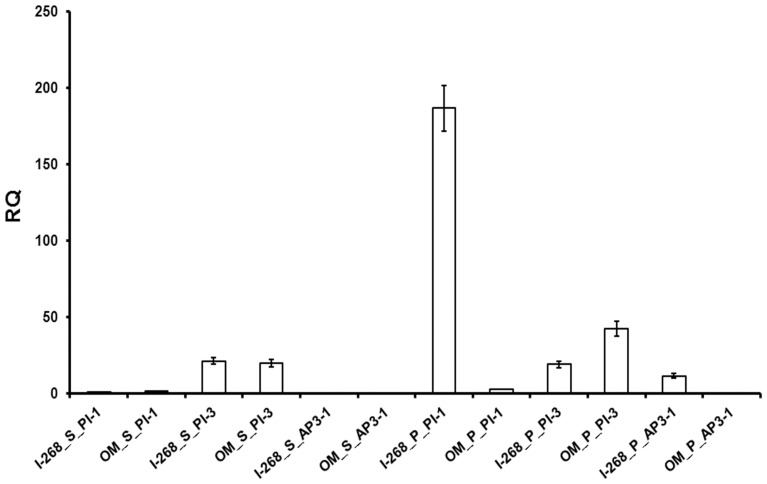
Comparison of quantitative expression levels of *PapsPI-1*, *PapsPI-3* and *PapsAP3-1* in the petals and sepals of the genotypes, I-268 and OM. The Y-axis represents relative quantities equilibrating the expression of *PapsPI-1* in 1-268 sepal as 1RQ value. Data represent mean + standard error of 3-5 biological replicates. On the X axis, the names of the genotypes (I-268 and OM) are followed by the organs (S: sepal; P: Petal), the gene expression (PI-1: *PapsPI-1* and PI-3: *PapsPI-3* and AP3-1: *PapsAP3-1*).

All the F_1_ plants (both normal and reciprocal crosses) showed normal petal phenotype demonstrating the recessive nature of the typical mutant character (*OM*). This also indicated the absence of cytoplasmic control of the mutant trait (sepaloid petal). The segregation pattern of the F_2_ populations of both reciprocal crosses also provided a good fit of the monogenic Mendelian ratio (P≥0.80-0.70) for the normal wild type (I-268) and the mutant (OM) characters indicating that the mutant trait is controlled by a single recessive nuclear gene “*OM*” (**Table 1**). Interestingly, *PapsPI-3* expression was higher than *PapsPI-1* expression in the normal sepals of I-268 and the sepaloid petal of OM, whereas in the normal petal (of I-268) *PapsPI-1* expression was higher than *PapsPI-3* expression. *PapsPI-1* expression in the normal petal was higher than that in the sepal (**Figure 8**). Relative expression of *PapsPI-1* in the sepal and sepaloid petal (I-268 and OM, respectively) was comparable to the *PapsPI-1* expression in the sepal of I-14 and sepal or the petaloid-devoid sepal part of the partially petaloid sepal of Pps-1.

## Discussion

Previously, a spontaneous true breeding homeotic gene mutant Pps-1 with distinct partial petaloid sepals was detected in the population of downy mildew (DM)–resistant elite genotype I-14 during identification of disease resistance sources in opium poppy at CSIR-CIMAP [20]. Analysis of this genotype clearly indicated single, recessive, nuclear gene control of the mutant character and demonstrated that the mutant phenotype is due to mutations at the *Pps-1* locus with a negative control function. In this investigation, a homeotic mutant (OM) was detected in the open-pollinated population of the genotype I-268 of opium poppy in which the petal morphology displayed sepal-like characteristics. This mutant was maintained by several selfing cycles and was observed to be controlled by a recessive mutation.

Expression of genes related to organ identity was measured in the two mutant genotypes, Pps-1 and OM, and compared with their parents, I-14 and I-268, respectively. When sepals of I-14 and Pps-1 genotypes were analyzed, only two genes (*PapsPI-1* and *PapsAP3-1*) showed maximal differential expression. *PapsAP3-1* and *PapsPI-1* have previously been shown to have high expression in petals [16]. Due to significant differential expression in partially petaloid sepals as compared to normal sepals, *PapsPI-1* was taken up for detailed study. As expected, the *PapsPI-1* expression was very high in petals of both I-14 and Pps-1. This is expected as *AP3* and *PI* gene products are believed to form a heterodimer that acts *in vivo* as part of a larger MADS box protein complex, specifying petal as well as stamen identity [8], [9], [11], [30], [31], [32]. In opium poppy, *PapsAP3-1* was able to heterodimerize with *PapsPI-1* like in *Arabidopsis* and *Antirrhinum* [7], [16], [33]. Although *PapsPI-1* is required to specify petal as well as stamen identity, *PapsAP3-1* functions primarily in the specification of petals and *PapsAP3-2* functions primarily in the specification of stamens [16]. Accordingly, we could observe the differential expression of *PapsAP3-1* in the partially petaloid sepals of Pps-1 genotype compared to the normal sepals of I-14. This confirms the involvement of *PapsPI-1* and *PapsAP3-1* in petal development in *P. somniferum*.

Further, to find out the role of *PISTILLATA* in the petaloid and normal (petaloid-devoid) part of the partially petaloid sepal, the expression of *PapsPI-1* and *PapsPI-3* was compared in petals, sepals, the petaloid portion of the partially petaloid sepals and normal (petaloid-devoid) part of the partially petaloid sepals. Two *PI* gene paralogues (*PapsPI-1* and *PapsPI-*3) were detected in this investigation instead of *PapsPI-1*and *PapsPI-2* as reported earlier [16]. The nucleotide sequence of *PapsPI-3* is different from both *PapsPI-1* and *PapsPI-2*. The expression of *PapsPI-3* was also observed to be always higher when compared to *PapsPI-1* in the normal sepal of I-14 and the normal (petaloid-devoid) part of the partially petaloid sepal of Pps-1, whereas a significantly higher expression of *PapsPI-1* was observed compared to *PapsPI-3* in the petals of both the genotypes and in the petaloid part of the partially petaloid sepal of Pps-1. As described earlier, protein produced by the gene *PapsPI-2* does not dimerize with PapsAP3-1, PapsAP3-2, PapsPI-1 or PapsPI-2. However, this does not obviate the possibility of interaction with other MADS-box gene products that affect its function [16]. Hence the role of *PapsPI-3* cannot be ruled out in partially petaloid sepal character of the Pps-1 flowers.

Ectopic expression of *Antirrhinum Glo* (*GLOBOSA)* in tobacco leads to petaloid sepals, and ectopic expression of both *Def* (*DEFICIENS)* and *Glo* leads to the almost complete conversion of sepals to petals [34]. *Glo* and *Def* are *PISTILLATA* and *APETALA3* orthologs from *Antirrhinum majus.* Ectopic expression of a single homeotic gene, the *Petunia* gene *GREEN PETAL*, has also been described as sufficient to convert sepals to petaloid organs [35]. In this investigation, *PapsPI-1* was expressed ectopically in tobacco under the CaMV 35S promoter and of the twenty transgenic plants (that flowered) screened, only one was observed to be producing flowers with partially petaloid sepal character (**Figure 7**). When some of the transgenic plants were analyzed, all showed *PapsPI-1* gene integration as well as expression in sepals (**Figure S2**). But, the expression of *PapsPI-1* in the sepals of transgenic plant producing flowers with the partially petaloid sepal was highest. Hence, in the case of the mutant Pps-1 of *P. somniferum* the overexpression of *PapsPI-1* in the sepal leads to their conversion to a petal-like phenotype, as corroborated in part by ectopic expression of *PapsPI-1* in tobacco. However, considering the small difference in the relative quantity (RQ) values between the partially petaloid sepal phenotype (II: 5.712) and the transgenic plant having normal flowers (Ta: 5.378), other reasons responsible for the low frequency of *PapsPI-1* transgene phenotype in tobacco cannot be ruled out. It is possible that the very small difference in expression (about 6%) might be the tipping point for initiating a developmental switch. But without other lines showing this phenotype, this is only a speculation and it is also possible that the random insertion of the construct in “Ta” might have caused a gene disruption resulting in the phenotype unrelated to the expression of *PapsPI-1*. The analysis of the transgenic lines Ta, Tb and Tc, not showing the desired phenotype, preclude the possibility of any undesirable effect of the CaMV 35S promoter, which has been described in the past for not yielding desired phenotypes, especially for transcription factors expressed under its control [36]. One specific example of misexpression of a component of the flowering regulatory network is ectopic overexpression of the *LFY* gene from *Arabidopsis* [37]. Heterologous expression of transcription factors can also be negatively influenced by the species chosen for overexpression [38]. There may be several reasons for our observing very low frequency of abnormal phenotypes, but the occurrence of a petaloid sepal phenotype while overexpressing the *PapsPI-1* gene cannot be ruled out. The *Pps-1* recessive mutation described earlier [20], was confirmed in this investigation to be controlling the expression of *PapsPI-1* and *PapsAP3-1*, which is higher in petals of both the plants as well as in the petaloid part of Pps-1 sepals compared to sepals of I-14 and normal (petaloid-devoid) part of the mutant Pps-1 sepals. The functional significance of the heterodimer formed by PapsAP3-1 and PapsPI-1 in determining the petal structure [16] cannot be ignored and in this analysis we observed overexpression of *PapsAP3-1* and *PapsPI-1* in the petaloid part as compared to the normal (petaloid-devoid) part of the sepal in Pps-1. Genes encoding products that function as key regulatory components, such as transcription factors, as well as those participating in large multi-protein complexes (e.g. MADS-domain proteins) [29], appear to be preferentially maintained owing to the requirement for a stoichiometric balance with other components in the pathway [39], [40]. Hence, it seems that the recessive *Pps-1* locus might be influencing the expression of *PapsAP3-1* and *PapsPI-1* in sepals during development (**Figures 5** **,** **6**). As the proteins encoded by *PapsAP3-1* and *PapsAP3-2* can heterodimerize with *PapsPI-1*, but *PapsAP3-2* can also homodimerize [16], the role of *PapsPI-3* cannot be ruled out for petaloid conversion of sepal although this type of gene (*PapsPI-3*) has been described to be having limited role in petal morphology as compared to *PapsPI-1*.

Hose in Hose mutants of primrose and cowslip have been found to show dominant homeotic conversion of sepals to petals [41]. The demonstration that in some cases up-regulation of a single B-function MADS box gene can lead to the development of petaloid sepals is consistent with the inheritance of the *Hose in Hose* as a single dominant locus [41]. In contrast, the *CHORIPETALA* and *DESPENTEADO* mutants of *Antirrhinum* are inherited as recessive mutations, which also result in the conversion of sepals to petals [42]. In the present study, the mutations controlling up-regulation of *PapsAP3-1* and *PapsPI-1* in conversion of sepal to petal (*Pps-1*) and down regulation of *PapsAP3-1* and *PapsPI-1* in the conversion of petal to sepal (OM) were confirmed to be recessive in nature. In conclusion, this study indicates a differential pattern of regulation for flowering-related genes in various whorls.

## Supporting Information

Figure S1 **PCR screening of transgenic plant.**
(DOCX)

Figure S2 **Analysis of transgenic plants.**
(DOCX)

Table S1 **Primers used in this experiment.**
(DOCX)
