# Supplementary material for: Recessive Loci Pps-1 and OM Differentially Regulate PISTILLATA-1 and APETALA3-1 Expression for Sepal and Petal Development in Papaver somniferum
Source: PLoS One. 2014 Jun 30;9(6):e101272. doi: 10.1371/journal.pone.0101272 (PMC4076319; doi:10.1371/journal.pone.0101272)
Supplement: Figure S1 — PCR screening of transgenic plant. (DOCX) [file pone.0101272.s001.docx]

**Figure S1.** PCR screening of transgenic plant

Amplification of genomic DNA of, N: non transformed plant; I: Only vector transformed plant and II: tobacco plant transformed with *PapsP1-1* with *PapsP1-1* gene specific primers. M refers to molecular weight marker (Lambda EcoRI and Hind III digest). Only the transformed tobacco plant (II) shows amplification for *PapsP1-1* gene.


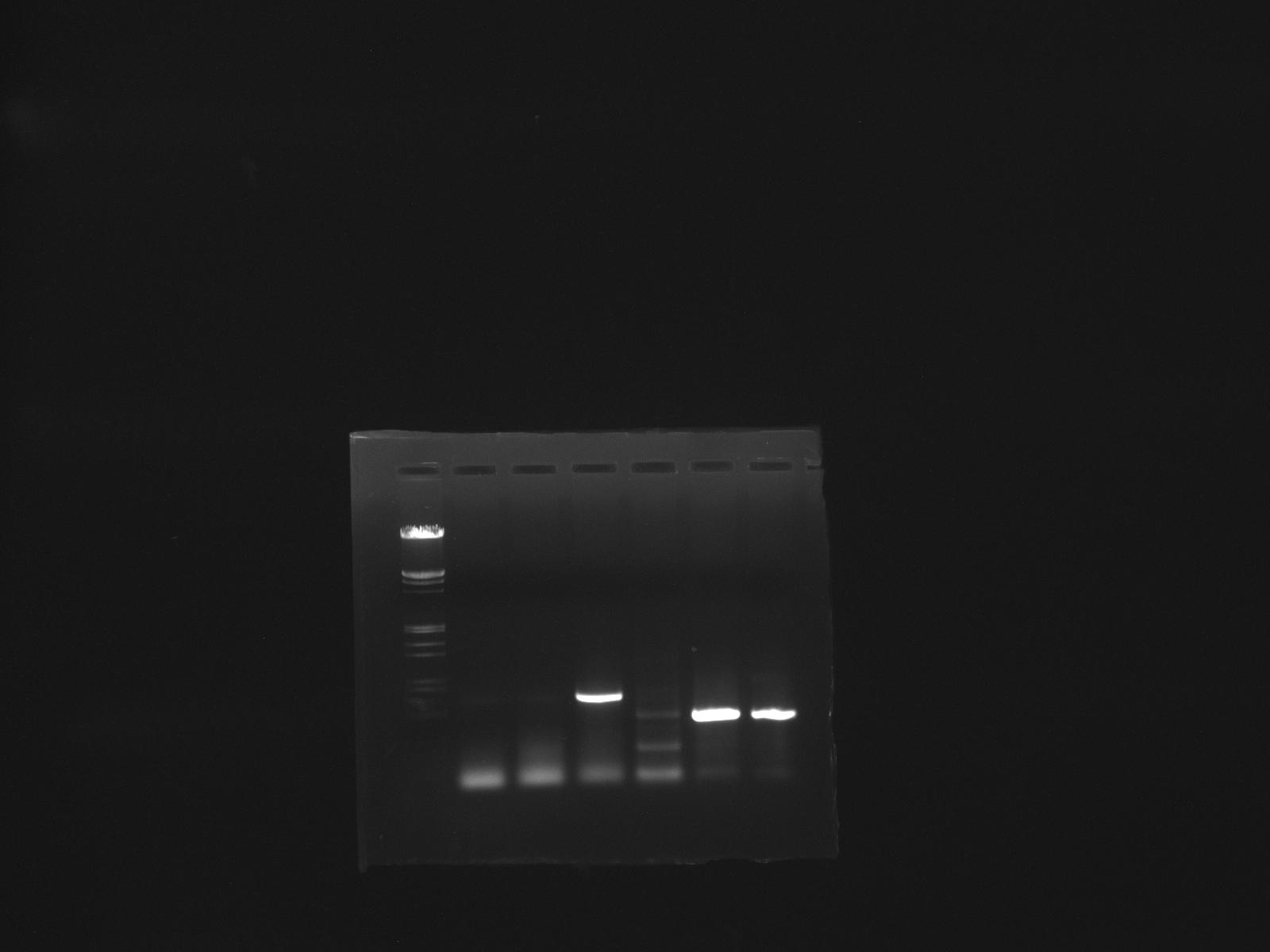


M N I II
