# Supplementary material for: Recessive Loci Pps-1 and OM Differentially Regulate PISTILLATA-1 and APETALA3-1 Expression for Sepal and Petal Development in Papaver somniferum
Source: PLoS One. 2014 Jun 30;9(6):e101272. doi: 10.1371/journal.pone.0101272 (PMC4076319; doi:10.1371/journal.pone.0101272)
Supplement: Figure S2 — Analysis of transgenic plants. (DOCX) [file pone.0101272.s002.docx]

**Figure S2.** Analysis of transgenic plants

A. Expression of *PapsPI-1* in the sepals of transgenic plants (V. Vector transformed; Ta to Tc and II are plants transformed with *PapsPI-1* gene). The Y-axis represents relative quantities equilibrating the expression of *PapsPI-1* in the sepals of vector transformed plants as 1RQ value. Data represent mean + standard error of 3-5 biological replicates.

B. Flower morphology of transgenic plants (the flowers are normal in all the transgenic lines except II which shows partially petaloid phenotype).

C. Amplification of genomic DNA shows the presence of *PapsPI-1* gene in all the transgenic plants.
