# Supplementary material for: Recessive Loci Pps-1 and OM Differentially Regulate PISTILLATA-1 and APETALA3-1 Expression for Sepal and Petal Development in Papaver somniferum
Source: PLoS One. 2014 Jun 30;9(6):e101272. doi: 10.1371/journal.pone.0101272 (PMC4076319; doi:10.1371/journal.pone.0101272)
Supplement: Table S1 — Primers used in this experiment. (DOCX) [file pone.0101272.s003.docx]

**Table S1: Primers used in this investigation**

**A. Primer sequences used to ascertain the genes expression through semi quantitative PCR.**

| **S. No.** | **Gene** | **Accession Number** | **Primer name** | **Primer Sequences (5’-3’)** |
| --- | --- | --- | --- | --- |
| **1** | ***PISTILLATA-1(PI-1)*** | *EF071994* | *PapsP1-1_F* | TTATCTTCTAGTACTGGG |
|  |  |  | *PapsP1-1_R* | TTAATCTAGTAGCTGGTATTG |
| **2.** | ***PISTILLATA-2(PI-2)*** | *EF071995* | *PapsP1-2_F* | GTTATGTTCTCTGAAGCTG |
|  |  |  | *PapsP1-2_R* | AGGACAAGCATATCCATCCATCT |
| **3.** | ***APETALA3-1(AP3-1)*** | *EF071993* | *PapsAP3-1_F* | TCTACTATGAAGGAGTTTTTC |
|  |  |  | *PapsAP3-1_R* | GATTGTACAAGTATCCTCCTC |
| **4.** | ***APETALA3-2(AP3-2)*** | *EF071992* | *PapsAP3-2_F* | AATGGAAATACAAAGAGGGTG |
|  |  |  | *PapsAP3-2_R* | TCAAGCAAGGCGAAGATA |

**B: Primer for cloning and quantitative RT-PCR analysis:**

| **S. No.** | **Gene** | **Accession Number** | **Primer name** | **Primer sequences (5’-3’)** |
| --- | --- | --- | --- | --- |
| **1.** | ***Actin*** | *EB740770* | *PapsAct_RT_F* | CACCTTCCAGCAGATGTGGAT |
|  |  |  | *PapsAct_RT_R* | AGCACTTACGGTGGACAATTGA |
| **2.** | ***Ubiquitin*** | *U66264.1* | *NtUb_RT_F* | ATGGAAGGACCTTGGCTGACT |
|  |  |  | *NtUb_RT_R* | GCACATCACGACCACAACCA |
| **3.** | ***PISTILLATA-1(PI-1)*** | *EF071994* | *PapsP1-1_RT_F* | ACCAGGGATTACCCGTCTCAT |
|  |  |  | *PapsP1-1_RT_R* | TGAATTGGCTGCATCTGGAA |
| **4.** | ***APETALA3-1(AP3-1)*** | *EF071993* | *PapsAP3-1_RT_F* | CAGTCTGGAGGCCAATTTGC |
|  |  |  | *PapsAP3-1_RT_R* | GGGAGCCAAGAACATGAAACTT |
| ***PapsP1-1 and PapsP1-3 gene primers for specific gene expression analysis*** | | | | |
| **1.** | ***PISTILLATA-1(PI-1)*** | *KF550916* | *PapsP1-1_F* | ATGGGTAGAGGTAAGATTGAG |
|  |  |  | *PapsP1-1_R* | CTACGGTGCTCCTTAATTTT |
| **2.** | ***PISTILLATA-3(PI-3)*** | *KF550917* | *PapsP1-3_F* | ATGGGTAGAGGTAAGATTGAG |
|  |  |  | *PapsP1-3_R* | CTACGGTGCTCCTTAATTT |
| ***PapsP1-1gene primers for pbi-121gene construct.*** | | | | |
| **1.** | ***PISTILLATA-1(PI-1)*** | *KF550916* | *PapsP1-1_F* | CTCTAGAATGGGTAGAGGTAAGATTGAG |
|  |  |  | *PapsP1-1_R* | GGGATCCTTAATCTAGTAGCTGGTATTTG |

**C. Primers for semi-quantitative RT-PCR analysis to check gene integration in transgenic plant.**

| **S.No.** | **Gene** | **Sequences** |
| --- | --- | --- |
| Kanamycin resistence gene | | |
| Forward primer | NPT II_F | ATCGGGAGCGGCGATACCGTA |
| Reverse primer | NPT II_R | GAGGCTATTCGGCTATGACTG |
| For amplification of PapsP1-1 gene | | |
| Forward Primer | PapsP1-1_F | ATGGGTAGAGGTAAGATTGAG |
| Reverse Primer | PapsP1-1_R | CTACGGTGCTCCTTAATTTT |
